# Prognostic Relevance of Methylenetetrahydrofolate Reductase Polymorphisms for Prostate Cancer

**DOI:** 10.3390/ijms17121996

**Published:** 2016-11-29

**Authors:** Victor C. Lin, Te-Ling Lu, Hsin-Ling Yin, Sheau-Fang Yang, Yung-Chin Lee, Chia-Chu Liu, Chao-Yuan Huang, Chia-Cheng Yu, Ta-Yuan Chang, Shu-Pin Huang, Bo-Ying Bao

**Affiliations:** 1 Department of Urology, E-Da Hospital, Kaohsiung 824, Taiwan; victorlin0098@yahoo.com.tw; 2 School of Medicine for International Students, I-Shou University, Kaohsiung 840, Taiwan; 3 Department of Pharmacy, China Medical University, Taichung 404, Taiwan; lutl@mail.cmu.edu.tw; 4 Department of Pathology, Kaohsiung Medical University Hospital, Kaohsiung 807, Taiwan; schoolyin@gmail.com (H.-L.Y.); sfyang@kmu.edu.tw (S.-F.Y.); 5 Department of Pathology, Faculty of Medicine, College of Medicine, Kaohsiung Medical University, Kaohsiung 807, Taiwan; 6 Department of Urology, Kaohsiung Medical University Hospital, Kaohsiung 807, Taiwan; leeyc12345@yahoo.com.tw (Y.-C.L.); m8201055@yahoo.com.tw (C.-C.L.); 7 Department of Urology, Faculty of Medicine, College of Medicine, Kaohsiung Medical University, Kaohsiung 807, Taiwan; 8 Department of Urology, National Taiwan University Hospital, College of Medicine, National Taiwan University, Taipei 100, Taiwan; cyhuang0909@ntu.edu.tw; 9 Department of Urology, National Taiwan University Hospital Hsin-Chu Branch, Hsinchu 300, Taiwan; 10 Division of Urology, Department of Surgery, Kaohsiung Veterans General Hospital, Kaohsiung 813, Taiwan; ccyu@vghks.gov.tw; 11 Department of Urology, School of Medicine, National Yang-Ming University, Taipei 112, Taiwan; 12 Department of Pharmacy, Tajen University, Pingtung 907, Taiwan; 13 Department of Occupational Safety and Health, China Medical University, Taichung 404, Taiwan; tychang@mail.cmu.edu.tw; 14 Graduate Institute of Medicine, College of Medicine, Kaohsiung Medical University, Kaohsiung 807, Taiwan; 15 Sex Hormone Research Center, China Medical University Hospital, Taichung 404, Taiwan; 16 Department of Nursing, Asia University, Taichung 413, Taiwan

**Keywords:** prostate cancer, radical prostatectomy, recurrence, genetic variation, methylenetetrahydrofolate reductase

## Abstract

Folate metabolism has been associated with cancers via alterations in nucleotide synthesis, DNA methylation, and DNA repair. We hypothesized that genetic variants in methylenetetrahydrofolate reductase (*MTHFR*), a key enzyme of folate metabolism, would affect the prognosis of prostate cancer. Three haplotype-tagging single-nucleotide polymorphisms (SNPs) across the *MTHFR* gene region were genotyped in a cohort of 458 patients with clinically localized prostate cancer treated with radical prostatectomy. One SNP, rs9651118, was associated with disease recurrence, and the association persisted after multivariate analyses adjusting for known risk factors. Public dataset analyses suggested that rs9651118 affects *MTHFR* expression. Quantitative real-time polymerase chain reaction analysis revealed that *MTHFR* expression is significantly upregulated in prostate tumor tissues when compared with adjacent normal tissues. Furthermore, overexpression of *MTHFR* correlates with cancer recurrence and death in two independent publicly available prostate cancer datasets. In conclusion, our data provide rationale to further validate the clinical utility of *MTHFR* rs9651118 as a biomarker for prognosis in prostate cancer.

## 1. Introduction

Accumulating evidence has suggested the involvement of folate status in modulating the risk of multiple cancers [1]. Folate can donate a methyl group to deoxyuridine monophosphate, converting it to thymidine monophosphate, which is used for DNA replication and repair. Folate deficiency can result in the compromised production of thymidine and misincorporation of uracil during cell division, leading to chromosomal instability. The enzyme methylenetetrahydrofolate reductase (MTHFR) catalyzes the irreversible reduction of 5,10-methylenetetrahydrofolate to 5-methyltetrahydrofolate, the predominant form of folate in plasma, which in turn provides the methyl group for the remethylation of homocysteine to convert to *S*-adenosylmethionine, the universal methyl group donor for numerous cellular methylation reactions including DNA methylation [2]. Low levels of folate may induce DNA hypomethylation and potentially activate oncogene transcription, leading to carcinogenesis [3].

Many studies have linked genetic variants in *MTHFR* to the risk of various types of cancer [4,5,6], including prostate cancer. A functional *MTHFR* gene polymorphism, rs1801133 (C677T), codes for an alanine to valine substitution at the folate binding site, and the enzyme activity for heterozygotes and variant homozygotes is reduced to approximately 60% and 30%, respectively, of that seen in wild-type homozygotes [7]. Meta-analyses have revealed that rs1801133 T might confer a protective effect against prostate cancer [4], but the prognostic effects of these variants on disease progression remain undetermined. Thus, we performed a systematic evaluation of common *MTHFR* gene variants in relation to the biochemical recurrence (BCR) after radical prostatectomy for patients with clinically localized prostate cancer.

## 2. Results

The clinical features of all participants are summarized in Table S1. After a median follow-up of 54 months, 184 (40.2%) patients had disease recurrence. BCR was significantly related to prostate-specific antigen (PSA) varieties at diagnosis, pathologic Gleason score, stage, surgical margin, and lymph node metastasis (*p* < 0.001).

**Figure 1:**
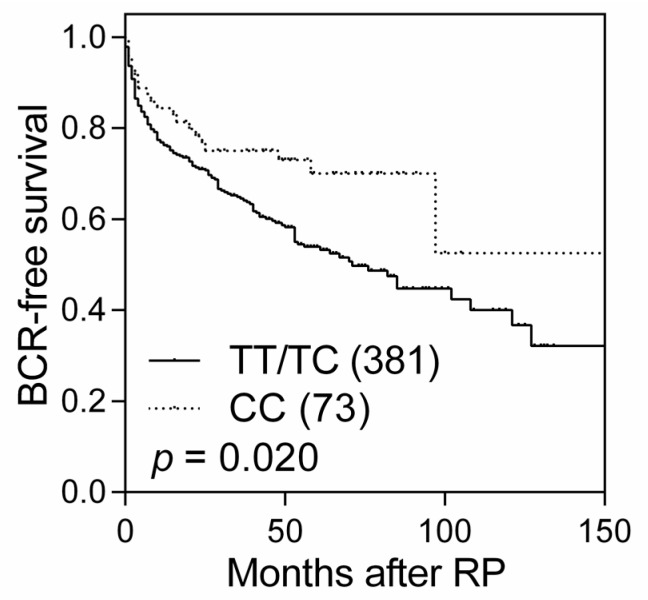
Kaplan-Meier curves comparing BCR-free survival by *MTHFR* rs9651118 genotypes. Numbers in parentheses indicate the number of patients. RP, radical prostatectomy.

Kaplan-Meier plots and log-rank tests were first used to assess the associations of *MTHFR* single-nucleotide polymorphisms (SNPs) with BCR in different genetic models (Table 1). A significant association was found between rs9651118 and BCR in the recessive model (*p* = 0.020). Patients with *MTHFR* rs9651118 CC genotype exhibited a 42% lower risk of recurrence (hazard ratio (HR) 0.58, 95% confidence interval (CI) 0.37–0.93, *p* = 0.023; Table 2 and Figure 1) when compared to those with TT and TC genotypes. The association persisted after adjusting for age, PSA at diagnosis, pathologic Gleason score, pathologic stage, surgical margin, and lymph node metastasis (HR 0.51, 95% CI 0.27–0.98, *p* = 0.044; Table 2).

**Figure 2:**
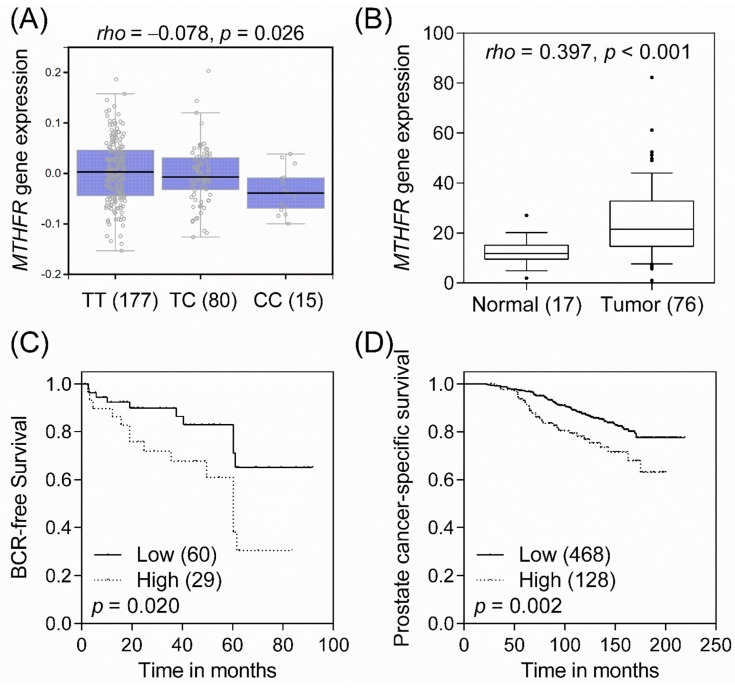
Functional analyses of *MTHFR* rs9651118 with prostate cancer progression: (**A**) Correlation of rs9651118 genotypes with *MTHFR* mRNA expression. Boxplot represents *MTHFR* mRNA expression according to the rs9651118 genotypes (GTEx dataset). There is a trend toward decreased *MTHFR* mRNA expression in the cells of rs9651118 C carriers; (**B**) *MTHFR* mRNA expression in 76 human prostate cancers and 17 adjacent normal tissue specimens, as determined by qRT-PCR, indicates that *MTHFR* is upregulated in the tumor tissues; (**C**) Kaplan-Meier analysis of BCR-free survival based on *MTHFR* mRNA expression in an independent set of prostate cancer microarray data [8]; (**D**) Kaplan-Meier analysis of prostate cancer-specific survival based on *MTHFR* mRNA expression in an independent set of public microarray data [9]. Numbers in parentheses indicate the number of patients.

In order to provide biologically plausible support for the observed associations, we initially used Genotype-Tissue Expression (GTEx) datasets to evaluate the association of rs9651118 with *MTHFR* expression. We observed that the rs9651118 T to C transition was negatively associated with *MTHFR* expression (Spearman’s rank correlation coefficient *rho* = −0.078, *p* = 0.026; Figure 2A), indicating that rs9651118 might be an expression quantitative trait loci for *MTHFR*. Subsequently, we examined differences in *MTHFR* gene expression between normal and cancer tissues. *MTHFR* gene expression was significantly higher in prostate tumor tissues compared to adjacent normal tissues (*rho* = 0.397, *p* < 0.001; Figure 2B). We further evaluated the prognostic value of *MTHFR* expression in prostate cancer progression using publicly available prostate cancer microarray datasets. Patients were dichotomized by *MTHFR* gene expression using an optimization algorithm for the minimum *p* value. High expression of *MTHFR* was associated with shorter BCR-free survival (*p* = 0.020; Figure 2C) and shorter prostate cancer-specific survival (*p* = 0.002; Figure 2D) in two independent datasets [8,9]. Taken together, the minor allele C of rs9651118 might decrease *MTHFR* expression and thus protect patients from cancer recurrence and death.

## 3. Discussion

In the present study, we investigated the clinical relevance of genetic variants in *MTHFR* for prostate cancer recurrence after radical prostatectomy. We showed that inheritance of a *MTHFR* rs9651118 CC genotype was associated with increased BCR-free survival. These results might be biologically credible, since cells carrying the C allele tend to have decreased expression of *MTHFR*. Validation of our initial findings in two independent cohorts further confirms that higher levels of *MTHFR* are associated with prostate cancer development and poorer patient outcomes.

The SNP rs9651118 is located in the intron 2 of *MTHFR*, and has low linkage disequilibrium with rs1801133 and rs3753582 (*r*^2^ < 0.30). Functional annotation using HaploReg revealed that rs9651118 coincides with regions of open chromatin, which probably correspond to the promoters or enhancers of *MTHFR* (Table S2). Specifically, the protective allele C is predicted to destroy a putative binding site for E1A binding protein p300 (EP300), which might result in lower *MTHFR* expression. EP300 is a transcriptional coactivator and has histone acetyltransferase activity favoring transcription via chromatin remodeling [10]. It has been shown that EP300 is upregulated by androgen ablation, and its expression correlates with worse prognosis in prostate cancer [11]. Similar to our results, a study reported that rs9651118 C was associated with reduced risk of lung cancer [12] and better survival of breast cancer [13]. The protective effects of rs9651118 C could be due to the decreased expression of *MTHFR*, resulting in increased availability of 5,10-methylenetetrahydrofolate and prevention of uracil misincorporation by aiding thymidine biosynthesis. Additionally, Kang and colleagues demonstrated that individuals carrying low enzyme activity of *MTHFR* polymorphisms showed a reduced level of aberrant hypermethylation in the promoter region of *O*-6-methylguanine-DNA methyltransferase, a DNA repair gene protecting cells from the cytotoxicity from alkylating agents [14]. Although *MTHFR* rs1801133 (C677T) has been shown to be related to prostate cancer risk [4], it was not associated with disease recurrence in this study. The reasons for this need further investigation, but our results suggest that different pathways might be involved during prostate cancer development and progression. Thus, further functional characterizations are warranted to validate these genotype-phenotype correlations.

## 4. Materials and Methods

### 4.1. Patient Recruitment and Data Collection

A total of 458 patients with clinically localized prostate cancer following radical prostatectomy as initial therapy were recruited from National Taiwan University (Taipei, Taiwan), E-Da (Kaohsiung, Taiwan), Kaohsiung Medical University (Kaohsiung, Taiwan), and Kaohsiung Veterans General hospitals (Kaohsiung, Taiwan), as described previously [15,16,17,18]. Patient baseline characteristics and treatment outcomes were collected from medical records. BCR was defined as PSA values of 0.2 ng/mL or more after radical prostatectomy [19,20]. Written informed consent was obtained from all patients, and the study was approved by the Institutional Review Board of Kaohsiung Medical University Hospital (#KMUHIRB-2013132; 21 January 2014; Kaohsiung, Taiwan) in accordance with the approved procedures.

### 4.2. Single-Nucleotide Polymorphism (SNP) Selection and Genotyping

Genomic DNA was isolated from peripheral blood using the QIAamp DNA Blood Maxi Kit (Qiagen, Valencia, CA, USA), and was stored until use. We utilized a haplotype-tagging SNP approach to capture common genetic variations in the *MTHFR* gene region. Haplotype-tagging SNPs were selected from Han Chinese in Beijing and Southern Han Chinese 1000 genomes data [21] using the Haploview Tagger (Broad Institute, Cambridge, MA, USA)with pairwise tagging (minor-allele frequency ≥0.1 and *r*^2^ ≥ 0.8) [22]. We identified eight SNPs, which were genotyped using Agena Bioscience MassARRAY iPLEX system at the National Center for Genome Medicine, Taipei, Taiwan. Genotyping quality control was performed, and the concordance rate was 100% among 10 duplicated samples. Any SNP that failed the assay design, deviated from Hardy-Weinberg equilibrium (*p* < 0.05), or was below a genotyping call rate of 0.8, was removed (number, *n* = 5). Thus, three SNPs were included for further statistical analyses.

### 4.3. Human Tissue Complementary DNA (cDNA) Array and TaqMan Quantitative Real-Time Polymerase Chain Reaction (qRT-PCR) Analysis

The expression levels of *MTHFR* and *ACTB* (β-actin) were measured using the TissueScan human prostate cancer cDNA array II and III, including 17 normal and 79 prostate cancer samples (OriGene Technologies, Rockville, MD, USA). qRT-PCR was performed using pre-validated TaqMan gene expression assays (Applied Biosystems, Foster city, CA, USA), *MTHFR* (Hs01114487_m1) and *ACTB* (Hs01060665_g1), on an Applied Biosystems 7500 according to the manufacturer's instructions. Quantification of each sample was normalized to the expression levels of the housekeeping gene *ACTB*.

### 4.4. Statistical Analysis

Kaplan-Meier plots and log-rank tests were used to evaluate the significance of patient characteristics, genotypes, and *MTHFR* gene expressions in relation to BCR. Crude or adjusted HR and 95% CI for BCR were estimated by univariate or multivariate Cox regression with adjustment of age, PSA at diagnosis, pathologic Gleason score, pathologic stage, surgical margin, and lymph node metastasis, as previously described [16]. The trend of *MTHFR* gene expression among normal and tumor tissues was analyzed by Spearman correlation. Statistical analysis was performed using the Statistical Package for the Social Sciences version 22.0.0 (IBM, Armonk, NY, USA), and two-tailed *p* < 0.05 was considered statistically significant.

### 4.5. Bioinformatics Analysis

HaploReg v4.1 [23] was used to identify the regulatory potential of the SNP. GTEx data were used to correlate the relationships between rs9651118 and *MTHFR* gene expression in transformed human fibroblasts [24]. Publicly available transcriptomic datasets [8,9] were used to evaluate the association of *MTHFR* expression and prostate cancer progression.

## 5. Conclusions

To the best of our knowledge, this is the first report to systematically study the prognostic impact of *MTHFR* genetic variants in patients with prostate cancer. We chose the BCR end point based on serum PSA due to its clinical relevance. In a population of patients treated with radical prostatectomy and did not receive additional therapies before disease progression, the median actuarial time from BCR to metastasis was five years. Once metastatic prostate cancer develops, the median time to prostate cancer-specific mortality was less than five years [25]. A rising PSA is often the first indication of the development of progressive disease and triggers a change in therapy. However, considering the limited number of patients and the homogeneous Taiwanese population in this study, these findings should be interpreted with caution and need to be validated in larger independent cohorts. Nevertheless, we performed multivariate analysis to reduce the false-positive findings, which revealed that our results remained significant even after adjusting for known clinical outcome predictors. Moreover, rs9651118 affects *MTHFR* expression and the expression is significantly correlated with prostate cancer development and progression in independent studies. Our study has provided further support for the prognostic value of *MTHFR* genetic variants, and has revealed the importance of folate metabolism in prostate cancer recurrence.

## Figures and Tables

**Table 1:** Association between haplotype tagging SNPs in *MTHFR* and BCR in clinically localized prostate cancer patients treated with RP.

SNP ID	Location	Chromosome	Position	Alleles	MAF	*p*		
						Additive	Dominant	Recessive
rs3753582	Intron 1	1	11805485	T > G	0.136	0.971	0.463	-
rs9651118	Intron 2	1	11802157	T > C	0.404	0.096	0.555	**0.020**
rs1801133	Exon 5	1	11796321	C > T	0.300	0.535	0.538	0.751

Abbreviations: SNP, single-nucleotide polymorphism; *MTHFR*, methylenetetrahydrofolate reductase; BCR, biochemical recurrence; RP, radical prostatectomy; MAF, minor allele frequency. *p*-values for log-rank test. *p* < 0.05 is in boldface.

**Table 2:** Univariate and multivariate analyses of *MTHFR* rs9651118 and BCR after RP.

SNP Genotype	Patients, *n*	BCR, *n* (%)	5-Year BCR-Free Survival, %	HR (95% CI)	*p*	HR (95% CI) ^a^	*p* ^a^
rs9651118							
TT	163	66 (40.5)	54.6	1.00		1.00	
TC	218	94 (43.1)	53.3	1.03 (0.75–1.41)	0.846	1.00 (0.68–1.47)	0.997
CC	73	20 (27.4)	70.1	**0.59 (0.36–0.98)**	**0.041**	0.51 (0.26–1.02)	0.057
TC/CC vs. TT				0.91 (0.67–1.24)	0.559	0.89 (0.61–1.29)	0.537
CC vs. TT/TC				**0.58 (0.37–0.93)**	**0.023**	**0.51 (0.27–0.98)**	**0.044**
Trend				0.84 (0.68–1.04)	0.100	0.81 (0.62–1.07)	0.132

Abbreviations: *n*, number; HR, hazard ratio; CI, confidence interval; *p*, *p*-value; PSA, prostate-specific antigen. ^a^ Adjusted by age, PSA at diagnosis, pathologic Gleason score, pathologic stage, surgical margin, and lymph node metastasis. *p* < 0.05 are in boldface.

## Supplementary Materials

Supplementary materials can be found at www.mdpi.com/1422-0067/17/12/1996/s1.
