# Supplementary material for: Prognostic Relevance of Methylenetetrahydrofolate Reductase Polymorphisms for Prostate Cancer
Source: Int J Mol Sci. 2016 Nov 29;17(12):1996. doi: 10.3390/ijms17121996 (PMC5187796; doi:10.3390/ijms17121996)
Supplement: Supplementary file 1 [file ijms-17-01996-s001.pdf]

Supplementary Materials: Prognostic Relevance of Methylenetetrahydrofolate Reductase Polymorphisms for Prostate Cancer

Victor C. Lin, Te-Ling Lu, Hsin-Ling Yin, Sheau-Fang Yang, Yung-Chin Lee, Chia-Chu Liu, Chao-Yuan Huang, Chia-Cheng Yu, Ta-Yuan Chang, Shu-Pin Huang and Bo-Ying Bao

Table S1. Clinical characteristics of study cohort.

Characteristic	*n* (%)	*p* [a]
Patients, *n*	458	
Age at diagnosis		0.517
Median, *y* (IQR)	66 (61–70)	
≤66	241 (52.6)	
>66	217 (47.4)	
PSA at diagnosis		<0.001
Median, ng/mL (IQR)	11.1 (7.1–17.5)	
≤10	197 (44.9)	
>10	242 (55.1)	
Pathologic Gleason score, *n* (%)		<0.001
≤6	160 (35.3)	
>6	293 (64.7)	
Pathologic stage, *n* (%)		<0.001
T1/T2	303 (67.2)	
T3/T4/N1	148 (32.8)	
Surgical margin, *n* (%)		<0.001
Negative	241 (72.6)	
Positive	91 (27.4)	
Lymph node metastasis, *n* (%)		<0.001
Negative	433 (95.6)	
Positive	20 (4.4)	
Recurrence	184 (40.2)	
Median follow-up time [b], *mo* (95% CI)	54 (50–58)	

Abbreviations: *n*, number; *p*, *p* value; *y*, year; IQR, interquartile range; PSA, prostate-specific antigen; T, primary tumor; N, regional lymph nodes; *mo*, month; CI, confidence interval. [a] *p*-value was calculated by the log-rank test for recurrence; [b] Median follow-up time and 95% CIs were estimated with the reverse Kaplan-Meier method.

Table S2. Regulatory annotation of *MTHFR* rs9651118.

Chr	Pos	Allele	AFR Freq	AMR Freq	ASN Freq	EUR Freq	Variant Type	Promoter Histone Marks	Enhancer Histone Marks	DNase	Motifs Changed	Selected eQTL Hits
1	11802157	T/C	0.01	0.19	0.39	0.22	Intronic	ESC, ESDR, LNG, IPSC, FAT, STRM, BLD, MUS, SKIN, LIV, BRN, GI, ADRL, HRT, KID, OVRY, CRVX, BRST, VAS, BONE	ESDR, IPSC, ESC, BRST, BLD, BRN, PANC, THYM, GI, PLCNT, SPLN, LIV	HRT, BLD	Cdx2, Foxa, Foxd1, HDAC2, p300	5 hits

Abbreviations: Chr, chromosome; Pos, position; AFR, African; AMR, American; ASN, Asian; EUR, European; DNase, DNase hypersensitivity; eQTL, expression quantitative trait loci.
